# LncRNA—UCA1 enhances cell proliferation and 5-fluorouracil resistance in colorectal cancer by inhibiting miR-204-5p

**DOI:** 10.1038/srep23892

**Published:** 2016-04-05

**Authors:** Zehua Bian, Liugen Jin, Jiwei Zhang, Yuan Yin, Chao Quan, Yaling Hu, Yuyang Feng, Heyong Liu, Bojian Fei, Yong Mao, Leyuan Zhou, Xiaowei Qi, Shenlin Huang, Dong Hua, Chungen Xing, Zhaohui Huang

**Affiliations:** 1 Wuxi Oncology Institute, Affiliated Hospital of Jiangnan University, Wuxi, Jiangsu, 214062, China; 2 Department of Surgical Oncology, Affiliated Hospital of Jiangnan University, Wuxi, Jiangsu, 214062, China; 3 Department of Surgery, the Second Affiliated Hospital of Soochow University, Suzhou, 215004, China; 4 Department of Oncology, Affiliated Hospital of Jiangnan University, Wuxi, Jiangsu, 214062, China; 5 Department of Pathology, Affiliated Hospital of Jiangnan University, Wuxi, Jiangsu, 214062, China; 6 Institutes of Biomedical Sciences and Shanghai Cancer Center, Shanghai Medical College, Fudan University, Shanghai, 200032, China

## Abstract

Recent preliminary studies reported the *in vitro* tumor-promoting effects of long non-coding RNA urothelial carcinoma associated 1 (UCA1) in colorectal cancer (CRC). However, the *in vivo* functions and molecular mechanism of UCA1 in CRC remain unclear. Therefore, we investigated the detailed role and mechanism of UCA1 in CRC. We found that UCA1 was up-regulated in CRCs and negatively correlated with survival time in two CRC cohorts. Functional assays revealed the *in vitro* and *in vivo* growth-promoting function of UCA1 and revealed that UCA1 can decrease the sensitivity of CRC cells to 5-FU by attenuating apoptosis. Further mechanistic studies revealed that UCA1 could sponge endogenous miR-204-5p and inhibit its activity. We also identified *CREB1* as a new target of miR-204-5p. The protein levels of CREB1 were significantly up-regulated in CRCs, negatively associated with survival time and positively correlated with the UCA1 expression. The present work provides the first evidence of a UCA1-miR-204-5p-*CREB1*/*BCL2*/*RAB22A* regulatory network in CRC and reveals that UCA1 and CREB1 are potential new oncogenes and prognostic factors for CRC.

Colorectal cancer (CRC) is the third most prevalent cancer type and the third leading cause of cancer-related deaths worldwide^1^. The occurrence and progression of CRC is a multi-step process involving in the deregulation of multiple oncogenes and tumor suppressors^2^. Although great efforts have been made to understand the complicated pathogenesis of CRC and to improve its treatment, CRC remains a severe disease. Therefore, further precise mechanisms underlying CRC need to be understood, and novel diagnostic and prognostic biomarkers need to be discovered.

Long non-coding RNAs (lncRNAs) are classified as a new kind of non-coding RNA (ncRNA) that is more than 200 nucleotides in length with no protein-coding capacity^3^. LncRNAs play important roles in diverse biological processes, including embryonic development, cell growth and tumorigenesis, by regulating gene expression at the chromatin organization, transcriptional and post-transcriptional levels^4^. For example, HOTAIR is generally deregulated and can regulate chromatin dynamics and gene expression in several types of cancer, including CRC 5. MALAT-1 is up-regulated in CRC tissues and mediates the Wnt/β-catenin signalling pathway to promote CRC invasion and metastasis^6^. In addition, recent studies have identified several other lncRNAs with important regulatory roles in CRC, suggesting the key roles of lncRNAs in the development and progression of CRC.

Human urothelial carcinoma associated 1 (UCA1) was first reported to be over-expressed in bladder cancer and was suggested to serve as a biomarker for the diagnosis of bladder cancer^7^. UCA1 is highly expressed in bladder cancer, tongue squamous cell carcinomas, breast cancer, gastric cancer and CRC, suggesting that UCA1 may play a common important role in human cancers^8^,^9^,^10^,^11^,^12^,^13^. The expression of UCA1 is regulated by the transcription factors C/EBPα and Ets-2, and UCA1 overexpression promotes cancer progression by regulating different pathways, including PI3K, AKT and mTOR-STAT3 signal pathways^14^,^15^,^16^. UCA1 can also function as a competing endogenous RNA (ceRNA) in cancer cells by interacting with microRNAs (miRNAs), a type of regulatory ncRNA. For example, UCA1 can modulate breast cancer cell growth and apoptosis by downregulating the tumor suppressive miRNA miR-143 17. miR-1 plays a tumor suppressive role by binding and inhibiting UCA1 in bladder cancer^18^. In addition, up-regulated UCA1 contributes to the progression of hepatocellular carcinoma by inhibiting miR-216b and activating the FGFR1/ERK signalling pathway^19^. These studies suggest the important functions and complicated mechanisms of UCA1 in human cancers. Although recent preliminary studies have reported the *in vitro* tumor-promoting effects of UCA in CRC and suggested its potential prognostic role^12^,^13^, the mechanism of UCA1 in CRC remains to be elucidated.

In the present study, we demonstrated that UCA1 was up-regulated in CRC tissues and predicted poor prognosis in two independent CRC cohorts. Ectopic expression of UCA1 promoted the proliferation of CRC cells and increased the chemoresistance of CRC cells to 5-fluorouracil (5-FU). Silencing UCA1 expression inhibited CRC cell growth and increased 5-FU-induced apoptosis. Further mechanistic study revealed that UCA1 exerted its oncogenic function in CRC by competitively sponging and then inhibiting miR-204-5p, a key tumor suppressive miRNA in gastroenteric tumors as reported in our previous works^20^,^21^. Up-regulated target genes of miR-204-5p, *CREB1* (newly identified in this study), *BCL2* and *RAB22A,* mediated the functions of UCA1 in cell proliferation and apoptosis. In addition, our data revealed that *CREB1* is a new prognostic factor in CRC. The present work provides the first evidence of the regulatory network of UCA1, miR-204-5p and miR-204-5p target genes in CRC, suggesting that UCA1 and *CREB1* are potential new therapeutic targets and prognostic factors for CRC.

## Results

### UCA1 is up-regulated in CRC tissues and predicts poor prognosis in CRC patients

**Figure 1:**
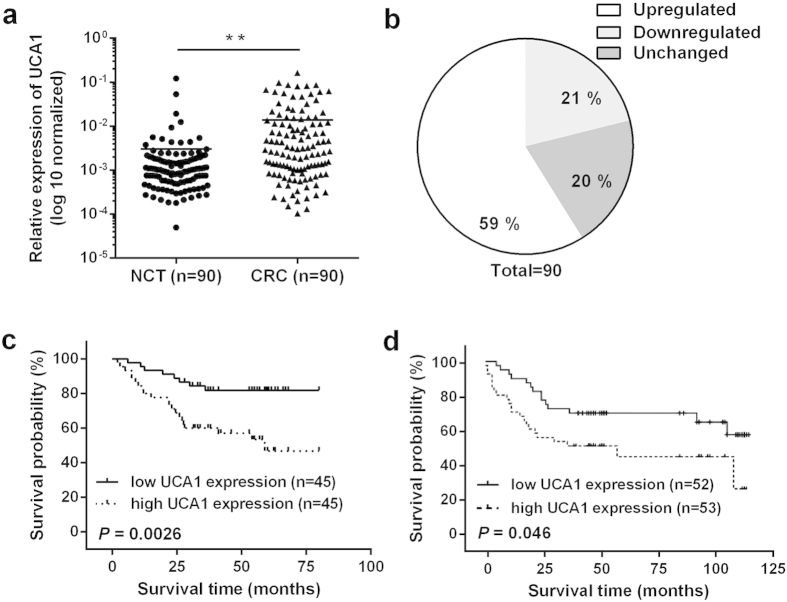
UCA1 is up-regulated and predicts poor prognosis in CRC. (**a**) UCA1 expression levels were analysed in 90 paired CRC and adjacent NCTs by qRT-PCR. UCA1 was obviously up-regulated in CRC tissues. (**b**) UCA1 was significantly up-regulated in 59% of CRC tissues compared with NCTs. (**c**) Kaplan-Meier overall survival curves for 90 patients with CRC classified according to relative UCA1 expression level. (**d**) Kaplan-Meier overall survival curves in another independent CRC cohort of 105 patients. **P < 0.01.

The levels of UCA1 were detected in 90 paired CRC tissues and their corresponding noncancerous tissues (NCTs) by quantitative reverse transcription-polymerase chain reaction (qRT-PCR), and >2-fold changes in UCA1 expression were designated as deregulated. The UCA1 expression was significantly up-regulated in 59% of (53 of 90) cancerous tissues compared with NCTs (*P* < 0.01) (Fig. 1a,b).

To correlate UCA1 expression with clinicopathologic features, the 90 CRC patients were classified into a relatively high group and a relatively low group using the median expression level of UCA1 in CRC tissues as a cut-off value. Noticeably, higher UCA1 expression in CRC was significantly correlated with a larger tumor size (*P* = 0.010), greater tumor depth (*P* = 0.041) and lymphatic invasion (*P* = 0.035) (Table 1).

To determine the potential relationship between UCA1 expression and the patients’ prognosis, Kaplan-Meier analysis and log-rank test were used to evaluate the effects of UCA1 expression on overall survival (OS). The results indicated that patients with higher UCA1 expression had a significantly poorer prognosis compared to patients with lower UCA1 expression (*P* = 0.0026) (Fig. 1c). To further assess whether the expression of UCA1 can be identified as a prognostic predictor for CRC patients, univariate and multivariate Cox proportional hazard analyses were performed. The analyses showed that UCA1 (HR = 2.395, 95% CI = 1.044–5.495, *P* = 0.039) and distant metastasis (HR = 3.004, 95% CI = 1.310–6.899, *P* = 0.009) were independent prognostic factors for CRC patients (Supplementary Table S1).

To confirm the prognostic value of UCA1, an independent CRC cohort (Supplementary Table S2) was recruited as a test group for survival analysis. As shown in Fig. 1d, higher UCA1 expression correlated with poorer survival time (*P* = 0.046). The up-regulation of UCA1 expression was also present in this independent CRC cohort (Supplementary Fig. S1).

### Identification of a new transcript of UCA1

Two different transcripts of UCA1 (~1.4 kb or ~2.3 kb) have been reported previously^7^,^8^. Two different PCR primers were used to amplify the full-length UCA1 transcript based on the two UCA1 transcripts (NR_015379.3 and GU799565) (Supplementary Fig. S2a), and only the ~1.4 kb transcript amplified. To test whether CRC cells express the ~2.3 kb UCA1 transcript, we designed PCR primers to amplify the sequence contained only in the ~2.3 kb transcript but not in the ~1.4 kb transcript. Consistent with the cloning result, the RT-PCR results showed no detectable expression of the ~2.3 kb transcript in CRC cell lines. Northern blotting analysis further confirmed the ~1.4 kb transcript as the major transcript of UCA1 (Supplementary Fig. S2b).

Interestingly, the cloned full-length cDNA of UCA1 was 1,456 bp in length, which included an additional 47 bp in the second exon compared with the reported UCA1 transcript sequences (NR_015379.3 and GU799565) (Supplementary Fig. S2c), indicating that this sequence is a new UCA1 transcript, which has been submitted to GenBank (KJ606608.1). To further confirm the results, we designed PCR primers based on the 3′ and 5′ terminal sequences near the inserted site in the UCA1 gene to test the existence of the 47 bp insert in several CRC cell lines. Two bands were observed, and the larger band was more abundant, indicating that the new transcript of UCA1 with the additional 47 bp is the primary splicing form of UCA1 in CRC cells (Supplementary Fig. S2d). The 1456 bp transcript of UCA1 was selected for subsequent functional studies.

### UCA1 increases CRC cell proliferation *in vitro* and *in vivo*

**Figure 2:**
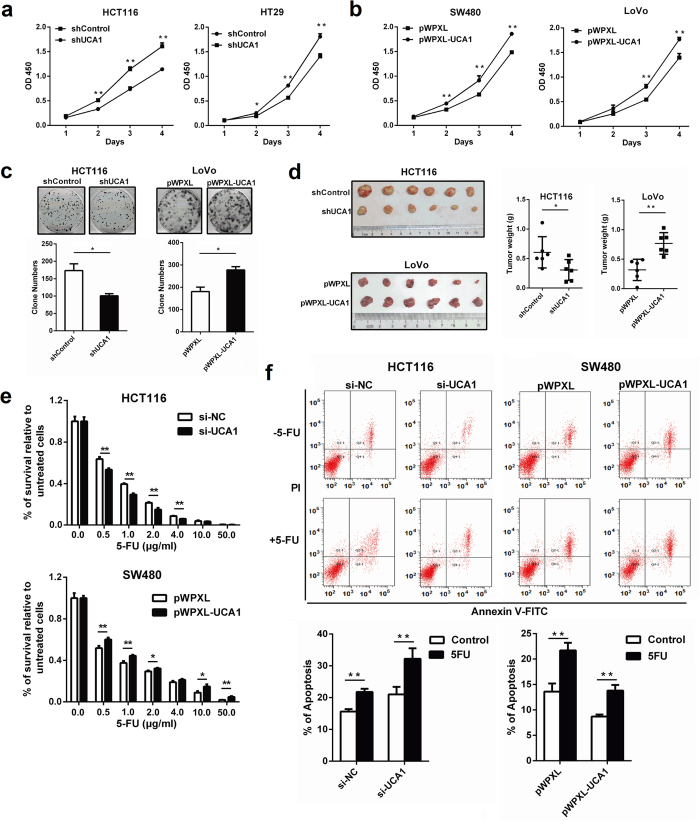
UCA1 promotes CRC cell growth *in vitro* and *in vivo*. (**a**,**b**) Effect of UCA1 on cell growth was detected by CCK-8 assay in HCT116/HT29 cells with silenced UCA1 and SW480/LoVo cells with overexpressed UCA1. (**c**) Effect of UCA1 knockdown and overexpression on colony formation of CRC cells. (**d**) Effect of UCA1 downregulation and overexpression on tumor growth in a xenograft mouse model. Overexpression of UCA1 promoted tumorigenesis, whereas silencing of UCA1 inhibited tumor growth. (**e**) HCT116 and SW480 cells transfected with siUCA1 or pWPXL-UCA1 were treated with 0 to 50 μg/mL 5-FU for 48 h, and cell viability was determined by CCK-8 assay. (**f**) Apoptosis of HCT116 and SW480 cells transfected with siUCA1/siNC or pWPXL-UCA1/pWPXL and treated with or without 0.2 μg/mL 5-FU was examined by flow cytometry. Representative scatter plots of PI vs Annexin V-FITC staining in one independent experiment. The values obtained from Annexin V assays represent the means ± S.D. for three independent experiments. *P < 0.05, **P < 0.01.

To select CRC cell lines for subsequent functional and mechanistic analyses of UCA1, qRT-PCR assays were performed to determine the relative UCA1 expression levels in different CRC cell lines. HCT116 and HT29 cells expressed relatively higher levels of UCA1, whereas SW480 and LoVo cells expressed relatively lower levels of UCA1 (Supplementary Fig. S3a). These cells were then used to establish cell lines with stable knockdown or overexpression of UCA1 (Supplementary Fig. S3b,c). A cell proliferation assay and colony formation assay revealed that silencing UCA1 expression significantly decreased cell proliferation, whereas overexpression of UCA1 promoted cell growth (Fig. 2a–c). Furthermore, down-regulation of UCA1 expression significantly inhibited the tumorigenicity of CRC cells, while overexpression of UCA1 promoted tumorigenicity in nude mice (Fig. 2d). No obvious effect of UCA1 on cell cycle was observed in CRC cells (Supplementary Fig. S4).

### UCA1 contributes to 5-FU resistance in CRC

Recent studies suggested the potential effect of UCA1 on chemoresistance in bladder cancer and ovarian cancer^22^,^23^; thus, we speculated that UCA1 might influence the response of CRC cells to the commonly used chemotherapeutic drug 5-FU. Cells with down-regulated and up-regulated expression of UCA1 were exposed to different concentrations of 5-FU. As expected, the survival rate of cells relative to the control was significantly lower in UCA1-depleted CRC cells and higher in UCA1-overexpressing cells (Fig. 2e), indicating that UCA1 can increase the resistance of CRC cells to 5-FU. Following treatment with 0.2 μg/mL 5-FU, significantly increased levels of apoptosis were observed in UCA1-depleted CRC cells, whereas significantly decreased apoptosis levels were found in UCA1-overexpressing CRC cells (Fig. 2f). Taken together, these data revealed that UCA1 can decrease the sensitivity of CRC cells to 5-FU by attenuating apoptosis.

### UCA1 sponges miR-204-5p

**Figure 3:**
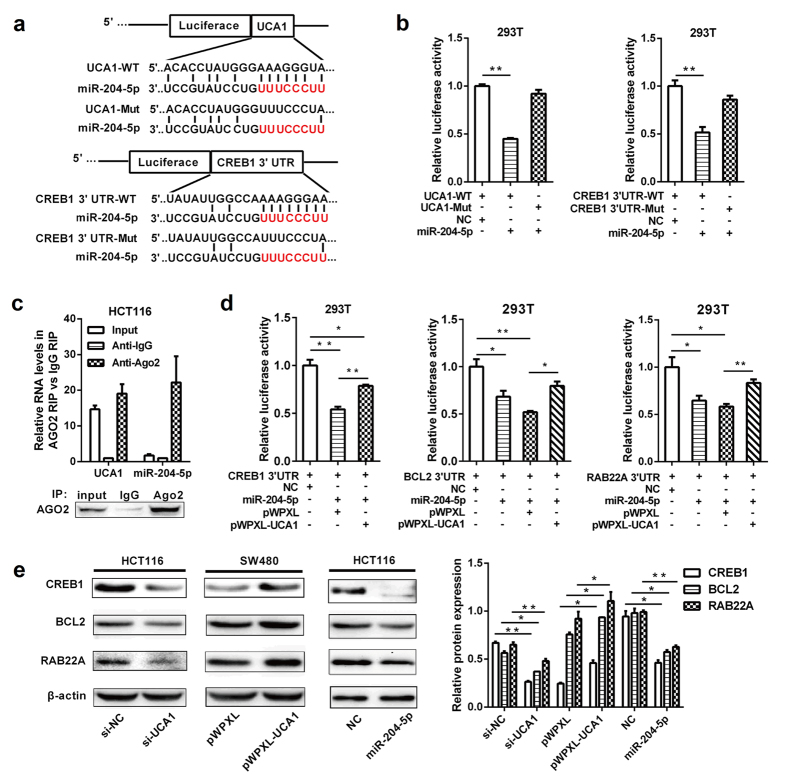
UCA1 inhibits the function of miR-204-5p and controls its target genes. (**a**) miR-204-5p binding sequence in UCA1 and *CREB1* 3′UTR. A mutation was generated in the UCA1 or *CREB1* 3′UTR sequences in the complementary site for miR-204-5p binding. (**b**) Luciferase activity of a luciferase reporter plasmid (pluc) containing wild-type or mutant UCA1/*CREB1* 3′UTR co-transfected with miR-204-5p was determined by the dual luciferase assay. (**c**) Cellular lysates from HCT116 cells were used for RIP with an Ago2 antibody and IgG antibody. The levels of UCA1 and miR-204-5p were detected by qRT-PCR. (**d**) miR-204-5p and pluc plasmids containing different 3′UTRs of the target genes of miR-204-5p were co-transfected with pWPXL-UCA1 or empty vector to verify whether UCA1 can function as a ceRNA of miR-204-5p. (**e**) Western blot analysis of the protein levels of miR-204-5p target genes (CREB1, BCL2 and RAB22A) in HCT116 cells transfected with siUCA1 or miR-204-5p mimics and in SW480 cells transfected with pWPXL-UCA1. *P < 0.05, **P < 0.01.

To elucidate the potential molecular mechanism of UCA1, we investigated the subcellular localization of UCA1 by qRT-PCR. The results showed that UCA1 is primarily located in the cytoplasm of CRC cells, suggesting that UCA1 may exert its regulatory function at the post-transcriptional level (Supplementary Fig. S3d). Based on previous reports that UCA1 functions as a ceRNA to regulate RNA molecules distributed primarily in the cytoplasm^17^,^18^,^19^, we presumed that UCA1 might promote CRC tumorigenesis by inhibiting miRNA function. A bioinformatics analysis revealed that UCA1 harbors a miR-204-5p recognition sequence, and miR-204-5p can decrease cell proliferation and the chemoresistance of CRC cells^21^ (Fig. 3a). Thus, we sought to confirm the potential association between UCA1 and miR-204-5p.

The sequence of UCA1 with a miR-204-5p binding site was inserted downstream of the luciferase gene to construct the pluc-UCA1 vector for the luciferase assay. The results showed that luciferase activity was significantly reduced in the wild-type UCA1 group compared with the vector control. To confirm that the reduction in luciferase activity of pluc-UCA1 was due to a direct interaction between miR-204-5p and UCA1, we mutated the miR-204-5p binding site in UCA1 (pluc-UCA1-Mut) and observed that the mutant UCA1 was completely refractory to miR-204-5p-mediated luciferase reporter repression (Fig. 3b), suggesting the sequence-specific binding of miR-204-5p to UCA1.

To test whether UCA1 and miR-204-5p are associated through miRNA ribonucleoprotein complexes (miRNPs), RNA immunoprecipitation (RIP) experiment was performed on the extracts of HCT116 cells using an Ago2 antibody. The qRT-PCR results showed that UCA1 and miR-204-5p were preferentially enriched in the Ago2-containing miRNPs compared with the control IgG immunoprecipitates (Fig. 3c). Thus, UCA1 is present in Ago2-contained miRNPs, likely through an association with miR-204-5p.

### UCA1 inhibits the function of miR-204-5p and controls its target genes

Although many target genes of miR-204-5p have been identified, we concentrated on *CREB1* because it is regulated by UCA1 and predicted to be a potential target gene of miR-204-5p by TargetScan^24^ (Fig. 3a). The wild-type and mutant 3′-untranslated region (UTR) of *CREB1* were independently cloned into the luciferase plasmid (pluc-*CREB1* 3′UTR-WT or 3′UTR-Mut) and co-transfected with miR-204-5p into HEK293T cells. The luciferase assay showed that miR-204-5p significantly inhibited the luciferase activity of the pluc-*CREB1* 3′UTR-WT reporter but not that of the pluc-*CREB1* 3′UTR-Mut reporter, suggesting that *CREB1* is a new target gene of miR-204-5p (Fig. 3b).

To further evaluate the relationship among UCA1, miR-204-5p and *CREB1*, pluc-*CREB1* 3′UTR-WT was co-transfected with miR-204-5p and pWPXL-UCA1 into HEK293T cells for a luciferase assay, and the results indicated that, in the presence of UCA1, the expression of the reporter gene was restored in the pluc-*CREB1* 3′UTR group compared with the control (Fig. 3d). This result indicates that UCA1 acts as an endogenous ‘sponge’ by binding miR-204-5p, thus abolishing the miRNA-mediated repressive activity on the *CREB1* 3′UTR. Furthermore, we demonstrated that UCA1 can affect the expression of the reporter gene in recombinant plasmids containing the 3′UTRs of *BCL2* and *RAB22A*, two other target genes of miR-204-5p identified in our previous work (Fig. 3d)^21^. In addition, the effects of UCA1 on the protein expression of miR-204-5p target genes (*CREB1*, *BCL2* and *RAB22A*) were determined in CRC cells using Western blot analysis. The results showed that both UCA1 knockdown and miR-204-5p overexpression induced a significant downregulation of the endogenous expression of these miR-204-5p target genes in HCT116 cells. In contrast, the protein expression of these targets was markedly up-regulated in UCA1-overexpressing CRC cells (Fig. 3e). Together, these data indicate that UCA1, by binding miR-204-5p, acts as a ceRNA of miR-204-5p to restore the expression of miR-204-5p target genes in CRC cells.

### CREB1 is overexpressed and positively correlates with UCA1 in CRC tissues

**Figure 4:**
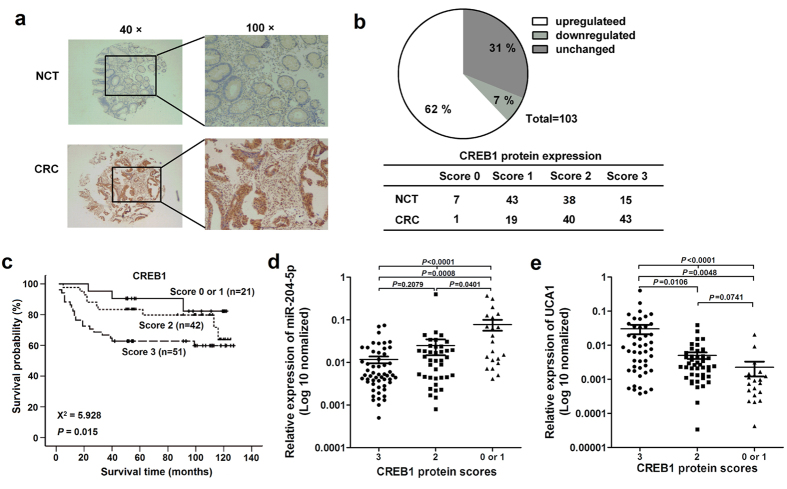
CREB1 is overexpressed in CRC and positively correlates with the expression of UCA1. (**a**) The protein expression of CREB1 was detected in 119 paired CRC tissues and NCTs by IHC, and CREB1 staining was available in 114 tumor tissues and 103 paired adjacent NCTs. (**b**) CREB1 protein expression was frequently increased in 103 CRC samples compared with the NCT samples. (**c**) Overall survival curves for 114 CRC patients classified according to the CREB1 expression levels in the tumor tissues. (**d**) Correlation analysis between CREB1 and miR-204-5p expression. (**e**) Correlation analysis between CREB1 and UCA1 expression.

In order to study the relationship between UCA1 and CREB1 in human CRC, we assessed CREB1 protein expression in CRC tissues by Immunohistochemistry (IHC) (Supplementary Fig. S5). As indicated in Fig. 4a,b, 64 of 103 (62%) tumors showed increased CREB1 expression compared with the paired NCTs. The results of survival analyses indicated that higher CREB1 protein levels were associated with poorer survival (*P* = 0.015, Fig. 4c). After adjusting for age, gender, TNM stage, tumor grade and size, Cox multivariate analyses showed that CREB1 expression is an independent prognostic factor for CRC (HR = 1.953; 95% CI = 1.125–3.389, *P* = 0.017). A correlation analysis showed that the expression of CREB1 was inversely correlated with the miR-204-5p levels (Fig. 4d) and was positively correlated with the UCA1 levels in CRC (Fig. 4e), suggesting that UCA1 acts as a ceRNA of miR-204-5p to regulate CREB1 expression in clinical CRC tumors.

### Role of miR-204-5p target genes in CRC is partly controlled by UCA1

**Figure 5:**
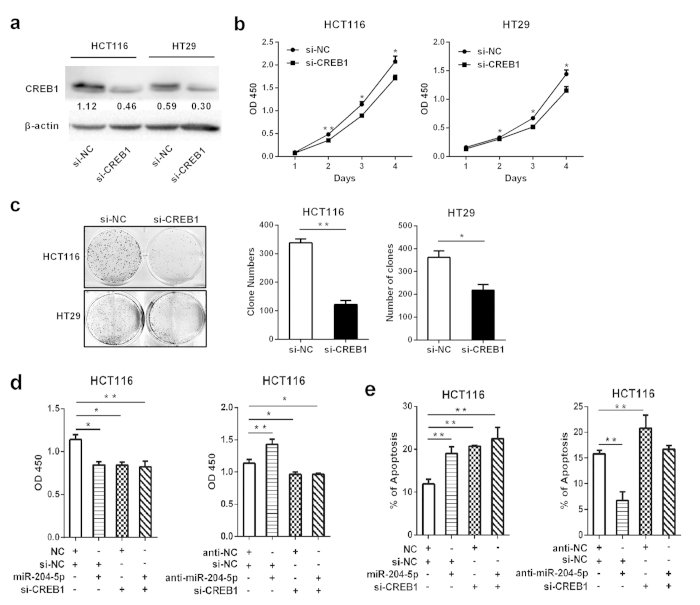
CREB1 promotes CRC cell proliferation and decreases cell apoptosis. (**a**) Western blot analysis was used to detect the effect of *CREB1* knockdown in HCT116 and HT29 cells. (**b**,**c**) CCK-8 assay and colony forming growth assay were performed to determine the proliferation and colony formation of CRC cells transfected with si*CREB1*. (**d**) The CCK-8 assay was used to determine the cell growth rate of CRC cells transfected with si*CREB1* and miR-204-5p mimics (or miR-204-5p inhibitor). (**e**) Silencing CREB1 expression prompted 5-FU-induced apoptosis. *P < 0.05, **P < 0.01.

**Figure 6:**
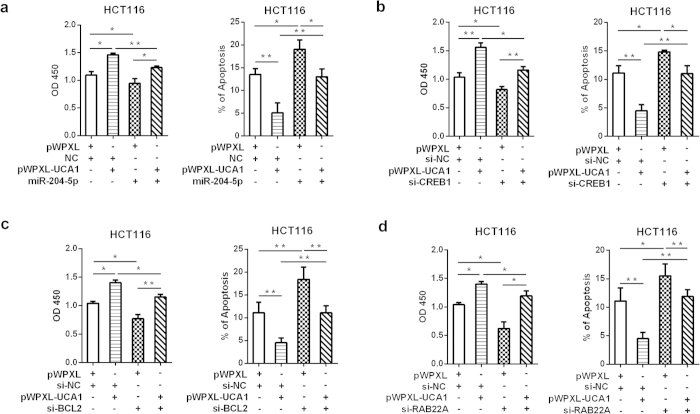
UCA1 controls the effects of miR-204-5p and its target genes on cell growth and apoptosis in CRC. (**a**) CCK-8 and apoptosis assays were used to detect cell growth and apoptosis in HCT116 cells transfected with miR-204-5p and pWPXL-UCA1. (**b**–**d)** CCK-8 and apoptosis assays were used to detect cell growth and apoptosis in HCT116 cells transfected with si*CREB1*, si*BCL2* or si*RAB22A* and pWPXL-UCA1. *P < 0.05, **P < 0.01.

To determine the functional role of *CREB1* in CRC, we first knocked down the expression of *CREB1* by siRNA (Fig. 5a). Cell proliferation assays and colony formation assays revealed that *CREB1* silencing decreased cell proliferation, suggesting the tumor-promoting function of *CREB1* (Fig. 5b,c). *CREB1* knockdown phenocopied the proliferation-repressing and apoptosis-inducing effects of miR-204-5p, whereas anti-miR-204-5p could not restore these effects in *CREB1*-silenced CRC cells (Fig. 5d,e), indicating that *CREB1* is a new functional target of miR-204-5p in CRC. In addition, in UCA1-overexpressing CRC cells, the ectopic expression of miR-204-5p counteracted the growth-promoting and apoptosis-inhibiting effects of UCA1, and silencing *CREB1* expression showed similar effects as miR-204-5p overexpression (Fig. 6a,b). Furthermore, silencing *BCL2* and *RAB22A* expression partly abrogated the growth-promoting and apoptosis-inhibiting effects of UCA1 (Fig. 6c,d). Collectively, these data indicated that the tumor-promoting role of the miR-204-5p target genes is controlled by UCA1.

## Discussion

Recent studies have suggested a key role for UCA1 in human cancers, mainly in bladder cancer. In this study, we found that UCA1 levels were much higher in CRC tissues than in their corresponding NCT tissues and negatively correlated with survival times. Functional and mechanistic studies revealed that UCA1 exerts its growth-promoting and apoptosis-inhibiting functions by acting as a ceRNA of miR-204-5p.

UCA1 was first reported in bladder cancer and showed extensive regulatory functions in cell proliferation, apoptosis, invasion, cell cycle, and drug resistance by different mechanisms^7^,^8^,^14^,^16^,^22^,^24^,^25^,^26^. Recent studies have also revealed that UCA1 dysregulation may play an important role in other human cancers^9^,^10^,^11^,^15^,^16^,^17^,^19^,^23^,^27^,^28^,^29^,^30^, including CRC 12,13. In this study, we confirmed the upregulation and prognostic value of UCA1 using different CRC cohorts^12^,^13^. In addition, we showed the *in vitro* and *in vivo* growth-promoting effects of UCA1 in CRC by a series of functional assays. UCA1 was reported to play positively or negatively regulating role on cell cycle by different researchers^13^,^17^, but we did not observe obvious effect of UCA1 on cell cycle of CRC cells. The controversial results may due to the difference in the UCA1 transcript or cell lines used in different studies.

UCA1 is located on chromosome 19p13.12 and has three exons. Two UCA1 transcripts of ~1.4 kb and ~2.3 kb were reported previously. Interestingly, we revealed that the ~1.4 kb transcript is the major form of UCA1 in CRC and identified a new UCA1 transcript of 1,456 bp with higher abundance than the previously reported transcripts. Han *et al.*^13^ reported that overexpression of the ~2.3 kb transcript of UCA1 had the same effects on cell growth and apoptosis as those of the ~1.4 kb transcript, suggesting that different transcripts of UCA1 may have similar functions.

Chemoresistance remains a major obstacle of tumor chemotherapy. Recent studies have showed that UCA1 increased cisplatin resistance in bladder cancer and ovarian cancer^22^,^23^ and may induce non-T790M-acquired resistance to EGFR tyrosine kinase inhibitors in EGFR-mutant non-small cell lung cancer, suggesting the important role of UCA1 in chemoresistance. 5-FU is a base analogue that acts as an anti-cancer drug primarily for digestive tract tumors. Although 5-FU is a basic chemotherapeutic drug for CRC, it is inefficient in most CRC patients due to drug resistance. Despite a great deal of efforts have been made to identify potential predictive markers of 5-FU, there is still a need of accurate markers to discriminate patients who are likely to benefit from 5-FU therapy^21^,^31^,^32^. In this study, we found that ectopic expression of UCA1 could promote the resistance of CRC cells to 5-FU by inhibiting 5-FU-induced apoptosis, whereas silencing UCA1 expression sensitized CRC cells to 5-FU by increasing apoptosis, suggesting that UCA1 may be a promising therapeutic target and predictive factor for the chemotherapy of CRC. In addition, 5-FU could affect cell cycle, but UCA1 did not influence the cell cycle of CRC cells in this study, which was consistent with our previous work on miR-204-5p^21^, suggesting that UCA1 or miR-204-5p influences 5-FU sensitivity mainly by inducing apoptosis instead of cell cycle arrest.

Recent studies have described an intricate interaction among different types of RNAs, including messenger RNAs and ncRNAs (such as lncRNAs, pseudogenes and circular RNAs). These RNA transcripts that contain miRNA binding sites can communicate with and co-regulate each other by competing for binding to shared miRNAs, thus acting as ceRNAs^33^,^34^. Although UCA1 was predicted to harbour the recognition sequence of many miRNAs and has been predicted to function as ceRNA of several miRNAs, including miR-1, miR-143 and miR-216b^17^,^18^,^19^, we focused on miR-204-5p because of its inverse functions compared with UCA1 in CRC as reported in our previous work^21^. As expected, a luciferase assay confirmed the direct binding of miR-204-5p to UCA1, and a RIP assay further supported the binding relationship of UCA1 and miR-204-5p in Ago2-containing miRNPs.

Of three key targets of miR-204-5p (*BCL2*, *RAB22A* and *CREB1*), *BCL2* and *RAB22A* have been reported to promote tumor growth and drug resistance, demonstrating the functional similarity of UCA1 21,35,36. *CREB1* is a well-known transcription factor and plays an oncogenic role during tumorigenesis and progression^37^. CREB1 is highly expressed in non-small cell lung cancer, breast cancer and gastric cancer, and correlates with a poor outcome of these cancers^38^,^39^,^40^. However, the expression, clinicopathological significance and mechanism of *CREB1* in CRC have not been reported. In this study, we found that *CREB1* was highly expressed in CRC and was verified to be a new target of miR-204-5p. Interestingly, UCA1 induces *CREB1* expression through the PI3K-AKT-dependent pathway in bladder cancer^24^, whereas *CREB1* was also suggested to increase *BCL2* expression^41^, suggesting a key role of *CREB1* in UCA1/miR-204-5p ceRNA signalling (Supplementary Fig. S6). Our data revealed that UCA1 up-regulated the target genes of miR-204-5p (*BCL2*, *RAB22A* and *CREB1*) by competitively sponging miR-204-5p, and thus promoted the proliferation and chemoresistance of CRC cells. The present work provides the first evidence for a ceRNA network that includes UCA1, miR-204-5p and miR-204-5p target genes in CRC cells.

In conclusion, we have identified the up-regulation of UCA1 in CRC and confirmed its prognostic value for CRC. UCA1 plays an important role in CRC tumorigenesis and progression by promoting cell proliferation, inhibiting apoptosis and inducing drug resistance through the UCA1/miR-204-5p axis. This correlation with a ceRNA allows us to better understand the function and mechanism of UCA1 in CRC development and progression and highlights UCA1 as a promising new target and prognostic factor for CRC treatment.

## Methods

### Cell lines and clinical samples

All cell lines, including HEK-293T, HCT8, HCT116, HT29, LoVo, and SW480, were purchased from the American Type Culture Collection (ATCC). All of the media (Hyclone, USA) were supplemented with 10% fetal bovine serum (Gibco, USA). All cells were maintained in a 5% CO_2_ cell culture incubator.

Two CRC cohorts, including 90 and 119 human primary CRC tissues and their paired adjacent NCTs, were collected at Fudan University Shanghai Cancer Center and the Affiliated Hospital of Jiangnan University, respectively (Table 1 and Supplementary Table S2). All of the patient materials were obtained with written informed consent from all subjects, and this project was approved by the Clinical Research Ethics Committees of Fudan University Shanghai Cancer Center and the Affiliated Hospital of Jiangnan University. All the methods in this study were in accordance with the approved guidelines and all the experimental protocols were approved by the Clinical Research Ethics Committees of Fudan University Shanghai Cancer Center and the Affiliated Hospital of Jiangnan University.

### RNA extraction and qRT-PCR analysis

Total RNA was extracted from cell or tissue specimens using RNAiso Plus (TaKaRa, Japan), nuclear and cytoplasmic RNA was extracted using PARIS^TM^ Kit (Ambion, USA). RNA was reverse-transcribed into cDNA using the PrimeScript^TM^ II 1st Strand Synthesis Kit (TaKaRa). Quantitative RT-PCR (qRT-PCR) was performed using UltraSYBR Mixture (CWBIO, China) in 96- or 384-well optical plates at 95 °C for 10 minutes, followed by 40 cycles of 95 °C for 15 seconds and 60 °C for 1 minute. After the reactions, the cycle threshold (Ct) data were determined using the default threshold settings. The relative gene expression levels were normalized to *β-actin* and calculated utilizing the 2^−ΔCt^ method. Stem-loop qRT-PCR assays using TaqMan miRNA probes (Applied Biosystems, USA) were performed to quantify the levels of the mature miRNAs.

### Vector constructs and siRNA

UCA1 was amplified from the cDNA of HCT116 cells using PrimerSTAR premix (TaKaRa) and cloned into the pWPXL lentivirus plasmid. The 3′UTR of *CREB1* and the UCA1 fragment containing the predicted potential miRNA binding sites were cloned into the luciferase reporter vector p-Luc; the mutant 3′UTR of *CREB1* and UCA1 carrying the mutated sequence in the complementary site for the seed region of miR-204-5p was constructed by overlap extension PCR as described in our previous work^42^. Primers for plasmid construction are listed in Supplementary Table S3. The shRNA sequence of UCA1 was also ligated into the pSIH-H1 vector. siRNAs of UCA1, *CREB1*, *RAB22A* and *BCL2* were purchased from GenePharma (China).

### Northern blotting

Northern blotting was performed using the NorthernMax^®^ Kit (Ambion) following the manufacturer’s protocol. Total RNA (20 μg) of HCT116 and HT29 cells mixed with formaldehyde load dye were separated by electrophoresis on 1% denaturing agarose-LE gel and then blotted onto nylon membranes (Life technologies, USA). The membranes were hybridized to the digoxin-labeled probe (Roche, USA) overnight at 68 °C. Hybridized RNA signals were detected using ImageQuant LAS 4000 mini after washing (GE, USA).

### Lentivirus production and transduction

The pWPXL, pWPXL-UCA1, pSIH-H1 or pSIH-H1-shUCA1 plasmid was co-transfected into HEK-293T cells along with the packaging plasmid ps-PAX2 and the envelope plasmid pMD2G using Lipofectamine 2000 (Invitrogen, USA). Virus particles were harvested 48 hours after co-transfection. The particles were then individually used to infect CRC cells to construct stable expression cell lines.

### Cell proliferation assay and colony formation assay

Cell viability was assessed by Cell Counting Kit 8 (CCK-8, Dojindo, Japan) according to the manufacturer’s instructions. For the colony formation assay, 800–1,500 cells were placed in each well of a 6-well plate and maintained in medium containing 10% FBS for 10 days. The colonies were fixed with methanol and stained with 0.1% crystal violet for 20 minutes, and the number of clones was counted using an inverted microscope.

### Assessment of chemotherapy sensitivity and apoptosis

CRC cells with UCA1 knockdown and overexpression were treated with 5-FU (range, 0–50 μg/mL), and cell inhibition was assessed by CCK-8 assay. For the apoptosis analysis, CRC cells were treated with 0.2 μg/mL 5-FU for 48 hours. The cells were then harvested and subjected to apoptosis analysis using an Annexin V-FITC and propidium iodide labelling kit (KeyGEN, China).

### Tumor formation in nude mice

CRC cells stably expressing UCA1, shUCA1 or control vector were subcutaneously injected into either flank of the same athymic male BALB/c nude mouse at 5 weeks of age. The mice were sacrificed after 4 to 6 weeks and examined for the growth of subcutaneous tumors.

### Luciferase reporter assay

HEK-293T cells were cultured in 96-well plates and co-transfected with 50 nM miR-204-5p mimic (or NC), 50 ng of luciferase reporter vector, and 5 ng of pRL-CMV using Lipofectamine 2000. Forty-eight hours after transfection, the luciferase activities were assayed using the Dual-Luciferase^®^ Reporter Assay System (Promega, USA).

### RIP assay

RIP was performed using the EZ-Magna RIP kit (Millipore, USA) following the manufacturer’s protocol. HCT116 cells at 80–90% confluency were collected and lysed in complete RIPA buffer. The whole cell protein extract was then incubated with RIP wash buffer containing magnetic beads conjugated with human anti-Ago2 antibody (Millipore) or mouse immunoglobulin G (IgG) control. The protein in the samples was digested with proteinase K, and the immunoprecipitated RNA was isolated. Finally, purified RNA was subjected to a qRT-PCR analysis to demonstrate the presence of the binding targets.

### Western blotting

The levels of CREB1, BCL2, and RAB22A were analysed by Western blotting using a rabbit polyclonal anti-human CREB1 (Proteintech, USA), BCL2 (Proteintech) or RAB22A (Abcam, USA) antibody at a dilution of 1:1,000, Normalization was performed by blotting the same samples with an antibody against β-actin (Beyotime, China).

### IHC

Tissue microarrays were constructed using paired CRC and NCT tissues. IHC staining was performed on 4-mm sections of paraffin-embedded tissue samples to detect the expression levels of CREB1 protein. In brief, the slides were incubated in CREB1 antibody diluted to 1:150 at 4 °C overnight. The subsequent steps were performed using the GTVisionTM III Detection System/Mo&Rb (Gene Tech, China).

### Statistical analyses

All results are presented as the mean values ± SEM. Student’s *t* test, the Mann-Whitney *U* test, the Kruskal-Wallis test and the χ2 test were used to determine the differences among different groups. The Kaplan-Meier method and log-rank test were conducted to determine differences in survival rates. A Cox proportional hazard analysis was used to evaluate the prognostic factors in univariate and multivariate analyses. A *P* value < 0.05 was considered statistically significant.

## Additional Information

**How to cite this article**: Bian, Z. *et al.* LncRNA—UCA1 enhances cell proliferation and 5-fluorouracil resistance in colorectal cancer by inhibiting miR-204-5p. *Sci. Rep.* **6**, 23892; doi: 10.1038/srep23892 (2016).

## Supplementary Material

Supplementary Information

## Figures and Tables

**Table 1:** Correlation of the expression of UCA1 with clinicopathologic features.

Characteristics	UCA1 expression		*P*value
	Low	High	
Ages (years)			
<60	20	28	0.093
≥60	25	17	
Gender			
Male	26	23	0.526
Female	19	22	
Tumor size (cm)			
<5	34	22	0.010
≥5	11	23	
Location			
Colon	26	21	0.292
Rectum	19	24	
Differentiation			
Well and moderately	39	35	0.274
Poorly	6	10	
Depth of tumor			
T1 + T2	13	5	0.041
T3 + T4	32	40	
Lymphatic invasion			
Absent	22	15	0.035
Present	23	30	
Distant metastasis			
Absent	41	37	0.223
Present	4	8	
Tumor stage			
I + II	23	14	0.056
III + IV	22	31	
