# Supplementary material for: LncRNA—UCA1 enhances cell proliferation and 5-fluorouracil resistance in colorectal cancer by inhibiting miR-204-5p
Source: Sci Rep. 2016 Apr 5;6:23892. doi: 10.1038/srep23892 (PMC4820696; doi:10.1038/srep23892)
Supplement: Supplementary Information [file srep23892-s1.doc]

Supplementary Information for

**LncRNA—UCA1 enhances cell proliferation and 5-fluorouracil resistance in colorectal cancer by inhibiting miR-204-5p**

Zehua Bian^1^, Liugen Jin^2,3^, Jiwei Zhang^1^, Yuan Yin^1^, Chao Quan^1^, Yaling Hu^1^, Yuyang Feng^1^, Heyong Liu^1^, Bojian Fei^2^, Yong Mao^1,4^, Leyuan Zhou^1,4^, Xiaowei Qi^5^, Shenlin Huang^6^, Dong Hua^1^, Chungen Xing^3^, Zhaohui Huang^1^,*

1. **Supplementary Table S1**. Univariate and multivariate regression analyses of parameters associated with prognosis of CRC patients
2. **Supplementary Table S2.** Patients’ information
3. **Supplementary Table S3**. Primer sequences
4. **Supplementary Figure S1**. UCA1 is up-regulated in an independent CRC cohort.
5. **Supplementary Figure S2**. A new transcript of UCA1 was identified in CRC cells.
6. **Supplementary Figure S3.** The effects of UCA1 overexpression and knockdown were verified in CRC cells.
7. **Supplementary Figure S4**. The effect of UCA1 on cell cycle distribution was determined by flow cytometry.
8. **Supplementary Figure S5**. Standard of immunohistochemical staining of CREB1 in the CRC and NCT samples.
9. **Supplementary Figure S6**. Diagram depicts the UCA1/miR-204-5p/CREB1, BCL2, and RAB22A ceRNA signalling axis in CRC.

**Supplementary Table S1. Univariate and multivariate regression analyses of parameters associated with prognosis of CRC patients**

| **Characteristics** | **Subset** | **Univariate analysis** | | **Multivariate analysis** | |
| --- | --- | --- | --- | --- | --- |
|  |  | ***P*** | **HR (95% CI)** | ***P*** | **HR (95% CI)** |
| Ages (years) | < 60/≥ 60 | 0.159 | 1.702 (0.813-3.566) |  |  |
| Gender | Male/Female | 0.183 | 0.595 (0.276-1.279) |  |  |
| Tumor size | < 5 cm/≥ 5 cm | 0.398 | 1.372 (0.659-2.853) |  |  |
| Location | Colon/Rectum | 0.932 | 1.032 (0.498-2.139) |  |  |
| Differentiation | Well + moderately/Poorly | 0.291 | 1.582 (0.676-3.704) |  |  |
| Depth of tumor | T1+T2/T3+T4 | 0.034 | 8.625 (1.173-63.423) | 0.219 | 3.551 (0.470-26.823) |
| Lymphatic invasion | Present/Absent | 0.000 | 13.443 (3.190-56.654) | 0.935 | 15537.752 (0.000-2.744E105) |
| Distant metastasis | Present/Absent | 0.000 | 5.630 (2.532-12.519) | 0.009 | 3.004 (1.310-6.889) |
| Tumor stage | I + II/III + IV | 0.001 | 12.723 (3.019-53.614) | 0.949 | 0.001 (0.000-8.935E97) |
| UCA1 | High/Low | 0.004 | 3.271 (1.444-7.408) | 0.039 | 2.395 (1.044-5.495) |

**Supplementary Table S2. Patients’ information**

| **Characteristics** | **Colorectal cancer**  **（n=119）** |
| --- | --- |
| Age (years) |  |
| <60 | 58 |
| ≥60 | 61 |
| Gender |  |
| Male | 64 |
| Female | 55 |
| Tumor size (cm) |  |
| <5 | 58 |
| ≥5 | 61 |
| Differentiation |  |
| G1 | 7 |
| G2 | 88 |
| G3 | 24 |
| Depth of tumor |  |
| T1 + T2 | 28 |
| T3 + T4 | 91 |
| Lymphatic invasion |  |
| Absent | 79 |
| Present | 40 |
| Tumor stage |  |
| I + II | 80 |
| III + IV | 39 |

**Supplementary Table S3. Primer sequences**

| **Primers** | **Sequences** | **Products length** |
| --- | --- | --- |
| **Primers for real time PCR** | | |
| UCA1-A | ATGTCCCAAGCCCTCTAAC |  |
| UCA1-S | ATGGTGTCCTCAAGCCTACT | 135 |
| UCA1-A2 | TACGGACATGCTTGACACTTG |  |
| UCA1-S2 | CGTAAGAGTTACCCGAAGCTC | 250/297 |
| UCA1-A3 | AGGAGCCAAGAAGTCTGGAG |  |
| UCA1-S3 | GTCATAATGGTGGAATGTCG | 103 |
| β-actin-F | AGTGTGACGTGGACATCCGCAAAG |  |
| β-actin-R | ATCCACATCTGCTGGAAGGTGGAC | 220 |
| **Primers for UCA1 cloning** | | |
| UCA1-FL | CGGGATCCTGACATTCTTCTGGACAATGAGTC |  |
| UCA1-FR | GGAATTCCTGACTCTTTTAGGAAGATTTCTTTTCTG | 1409/1456 |
| UCA1-FR2 | GGAATTCGGCATATTAGCTTTAATGTAGGTG | 2314 |
| **Pimers for construction luciferase reporter vectors** | | |
| UCA1-Y-F | CGGAATTCGACCAGCATCCAGGACAACA | 317 |
| UCA1-Y-R | CGGGATCCGCTTTAATGTAGGTGGCGATGA |  |
| CREB1-U-F1 | CTGGGAAGTTGGTGGTCCTC | 719 |
| CREB1-U-R1 | TGGTTGCCAATATGGTACTTGAG |  |
| CREB1-U-F2 | CGGAATTCATGCAAGAATGGTGGGGAGG | 380 |
| CREB1-U-R2 | CGGGATCCGGATCTAGCCCTTTCTAAGCCA |  |
| UCA1-M-R1 | GATGATACATTAGGGAAATCCCATAGGTGTGAGTGG |  |
| UCA1-M-F2 | ACACCTATGGGATTTCCCTAATGTATCATCGGCTTAGCA |  |
| CREB-M-R1 | ATGACATTTTTATAGGGAAATGGCCAATATAAAGAGAAAAACAT |  |
| CREB-M-F2 | TTATATTGGCCATTTCCCTATAAAAATGTCATCATAGGAATTTGTAC |  |
| F1 | GACGAAGTACCGAAAGGTCT |  |
| R2 | GCTGGCAACTAGAAGGCA |  |
| F3 | TCCTCATAAAGGCCAAGAA |  |
| R3 | CTAGCATTTAGGTGACACTATAGA |  |


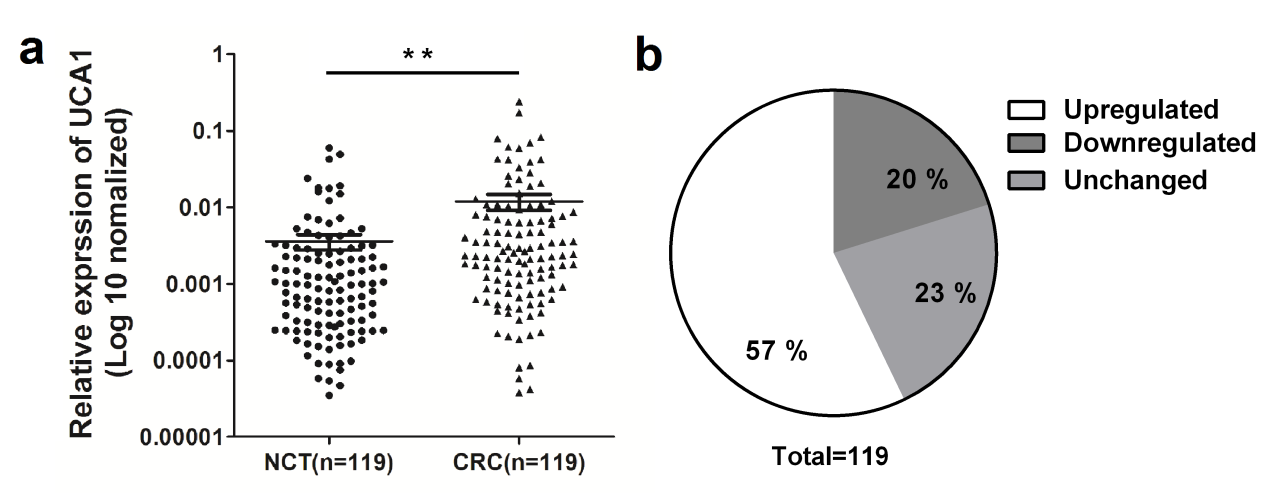


**Supplementary Figure S1. UCA1 is up-regulated in an independent CRC cohort. (a)** The expression level of UCA1 was analysed in an independent CRC cohort by qRT-PCR, and *β-actin* was used as an internal reference gene. **(b)** UCA1 was up-regulated in 57 % of 119 paired CRC tissues. ** *P* < 0.01.

**
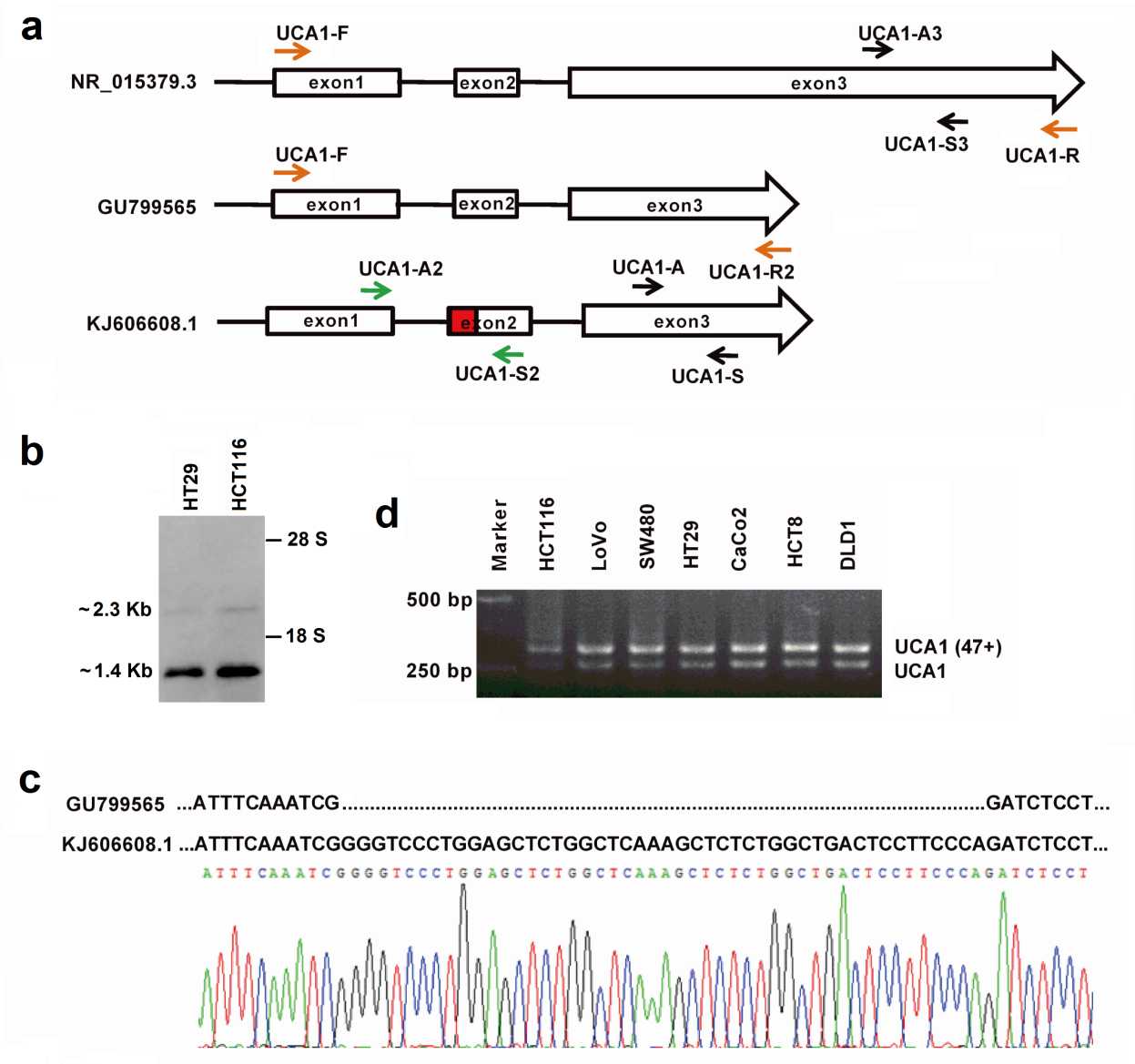
**

**Supplementary Figure S2. A new transcript of UCA1 was identified in CRC cells. (a)** The gene structure of the transcripts of UCA1 (NR_015379.3, GU799565 and KJ606608.1) and the primers used in this study are shown in this diagrammatic sketch. The orange arrows represent the primers used in UCA1 cloning; the black arrows represent the primers used in qRT-PCR; and the green arrows represent the primers used to verify the insert fragment. The red box represents the location of 47 bp in transcript KJ606608.1. **(b)** Northern blotting analysis of UCA1 expression in CRC cell lines. Two different splice variants of UCA1 (~1.4 kb and ~2.3 kb) were observed and the ~1.4 kb transcript was most abundant. **(c)** The sequencing result suggested the additional 47 base pairs in the full-length cDNA cloned from HCT116 cells compared with the reported UCA1 gene sequence (GU799565). **(d)** RT-PCR results showed that there are two products amplified from the 238^th^ base to the 487^th^ base of the UCA1 cDNA sequence using primers UCA1-A2 and UCA1-S2. The longer product with the 47 bp was the primary transcript.

**
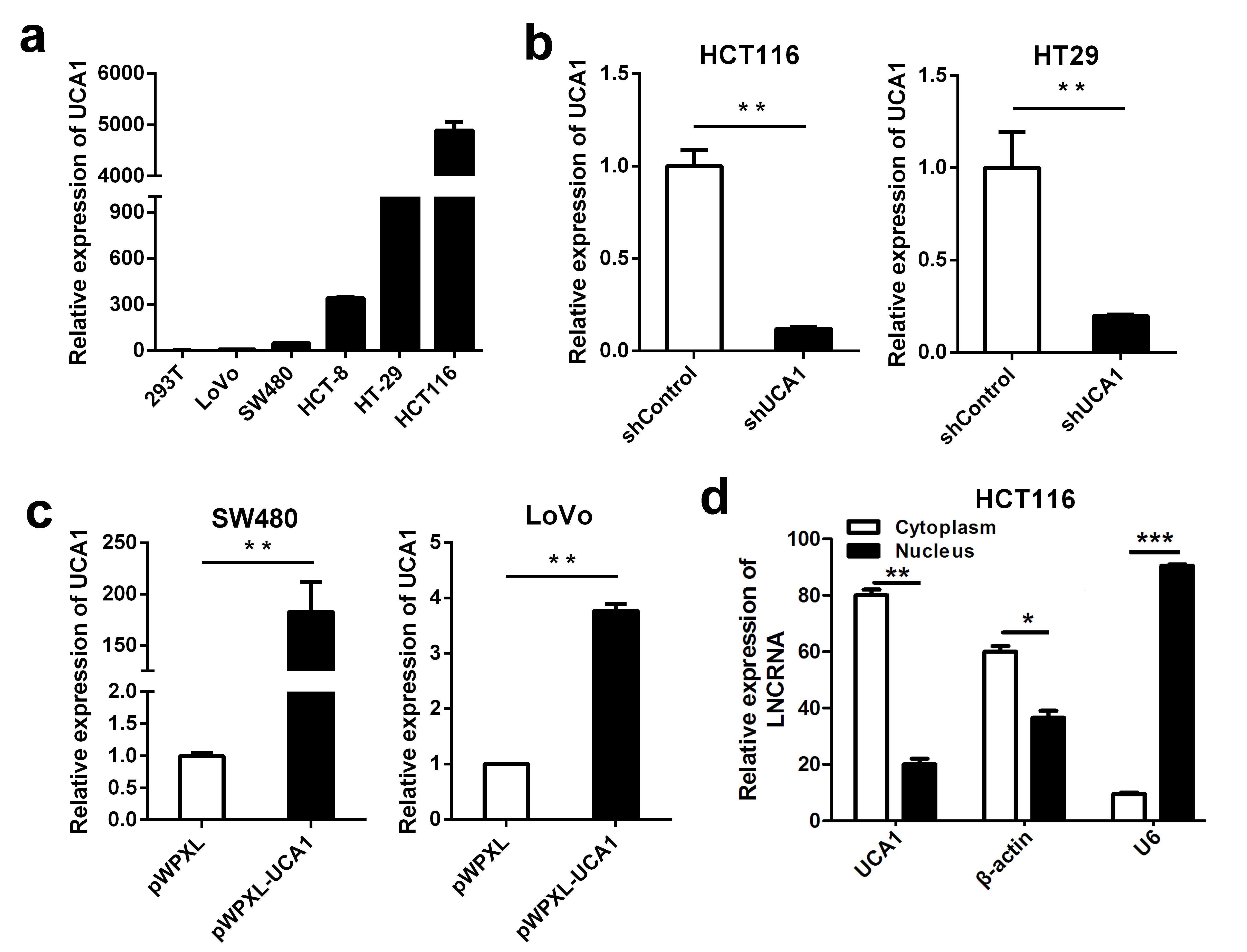
**

**Supplementary Figure S3. The effects of UCA1 overexpression and knockdown were verified in CRC cells. (a)** The expression levels of UCA1 in different CRC cell lines were determined by qRT-PCR. **(b-c)** The effects of UCA1 knockdown in HCT116/HT29 cells and over-expression in SW480/LoVo cells were examined by qRT-PCR. **(d)** Sub-cellular localization of UCA1 was analysed by qRT-PCR. U6 snRNA and β-actin were used as controls. * *P* < 0.05, ** *P* < 0.01.

**
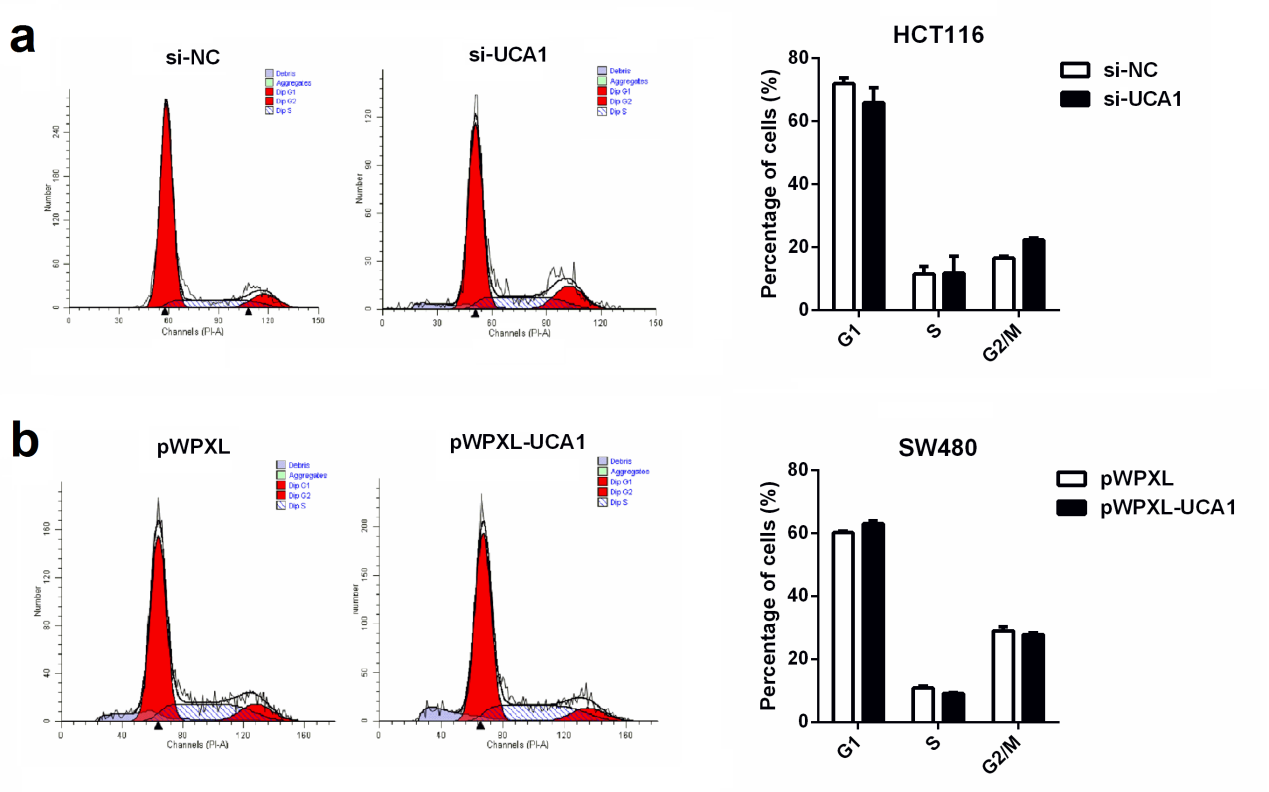
**

**Supplementary Figure S4. The effect of UCA1 on cell cycle distribution was determined by flow cytometry. (a) and (b)** Cell cycle analyses were performed in HCT116 cells transfected with siUCA1 or SW480 cells transfected with pWPXL-UCA1.

**
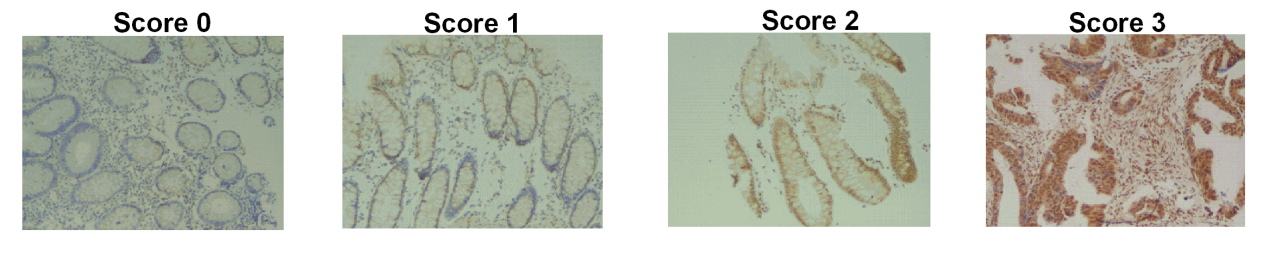
**

**Supplementary Figure S5. Standard of immunohistochemical staining of CREB1 in the CRC and NCT samples.** The scores (0, 1, 2 and 3) of CREB1 protein are based on the intensity of the brown staining.

**
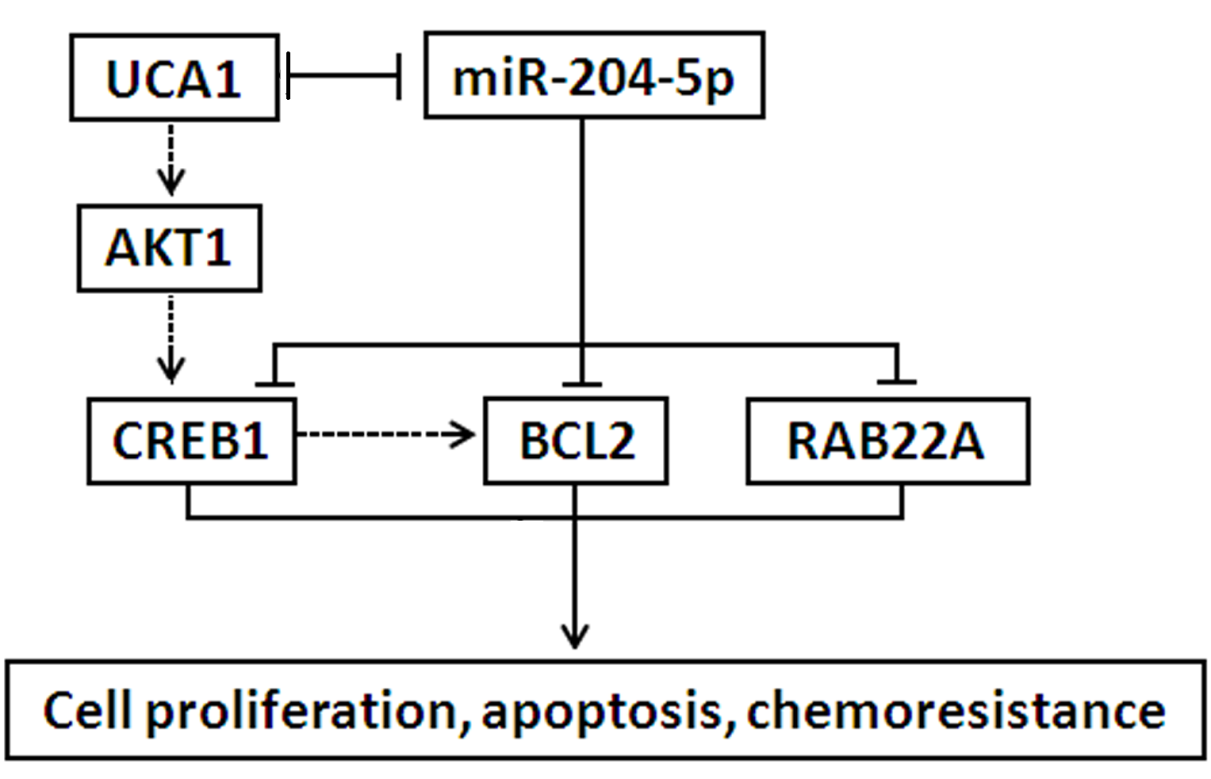
**

**Supplementary Figure S6. Diagram depicts the UCA1/miR-204-5p/CREB1, BCL2, and RAB22A ceRNA signalling axis in CRC.** The dotted lines represent the association between genes confirmed by other researchers.
